# Assessment and prevalence of concomitant chemo-radiotherapy-induced oral mucositis in patients with oral squamous cell carcinoma

**DOI:** 10.3906/sag-2007-131

**Published:** 2021-04-30

**Authors:** Sadia MINHAS, Aneequa SAJJAD, Rabia Mushtaq CHAUDHRY, Hamza ZAHID, Azfar SHAHID, Muhammad KASHIF

**Affiliations:** 1 Department of Oral Pathology, Akhtar Saeed Medical and Dental College, Lahore Pakistan; 2 Department of Oral Medicine, Akhtar Saeed Medical and Dental College, Lahore Pakistan; 3 Department of Oral Pathology, Bakhtawar Amin Medical and Dental College, Multan Pakistan

**Keywords:** Oral cancer, oral mucositis, CCRT, OMAS scale, exfoliative cytology

## Abstract

**Background/aim:**

Quantification of oral mucositis that progresses during concomitant chemo-radiotherapy (CCRT) is essential for its management. It is important to determine the methods that are simple, reliable and beneficial in foreseeing mucositis at earlier stages of treatment.

**Materials and methods:**

A prospective study was conducted on 100 oral cancer patients receiving CCRT following the inclusion criteria. Patients were evaluated for mucositis i.e. erythema and ulcers by using the World Health Organization (WHO) scale and the oral mucositis assessment scale (OMAS), whereas mature and immature cells were identified by exfoliative cytology. Clinical examination and procedure of oral cavity were performed before, on days 5, 17, and at the end of treatment.

**Results:**

Oral mucositis was observed in all oral squamous cell carcinoma (OSCC) patients receiving CCRT on different days with noteworthy increase from day 5 of CCRT to the end of treatment. For OMAS grading related to ulceration and erythema, Grade 1 (7.2%; 34%) was most commonly seen on the 5th day of CCRT, Grade 2 (29%; 19%) and Grade 3 (19%) were most frequently seen at the 17th day and end of CCRT, accordingly. With respect to WHO scale grades 1 and 2 (18.3%; 21.5%) was most frequently observed at the 17th day of CCRT, whereas grades 3 and 4 (12.5%; 2%) was noted at the end of CCRT. There was statistically significant increase in the percentage of immature cells at the end of CCRT (99%). A significant association (P < 0.0000) was observed among the days of smear and maturation stages of epithelial cells as well as among WHO mucositis grading, OMAS and types of epithelial cells, respectively.

**Conclusion:**

According to the findings of the study, oral mucositis grade is directly proportional to the progressing days of CCRT. Oral mucositis is frequently related to adverse clinical outcomes, affecting the patient’s quality of life. It is essential to develop methods that can be employed for the assessment of CCRT associated oral mucositis.

## 1. Introduction

Oral mucositis, also known as stomatitis, is a usual, dose-limiting and possibly incapacitating complication of concomitant chemo-radiotherapy (CCRT) appearing in more than 90% of head and neck cancer patients [1]. It can either be caused by the systemic effects of chemotherapeutic drugs or as a result of direct damage to the oral mucosa in radiotherapy [2]. Aggressive regimens are now considered to be an effective method to arrest the tumour growth and increase the chances of survival of advanced head and neck cancer patients, leading to further possible complications [3]. These treatments unfortunately do not exclusively target neoplastic cells, but also equally affect the cellular homeostasis of normal host cells. This leads to disturbances in the function of many different cells, among which is also the actively dividing epithelial cells of the oral cavity [4]. The loss of these epithelial cells results in mucositis, which is manifested as mucosal atrophy, necrosis and ulceration, thus causing pain, difficulty in chewing and swallowing [5,6]. This fragile oral mucosa, when augmented with reduced immunity, puts patients at high risk of opportunistic infections in the mouth. Mucositis may also extend and involve the gingivae and teeth of the patients, raising hygiene, aesthetic and speech concerns. All of this collectively affects patients’ confidence, as well as quality of life [7].

Mucositis induced by CCRT is a dose-regulated and expensive side effect [8]. Mucositis starts to occur as early as 5 to 10 days after the start of treatment as a result of direct radiation or indirectly due to drug-induced neutropenia causing mucositis [9,10]. The extent of acute toxicity produced by CCRT is considerably higher than the radiotherapy or chemotherapy alone due to intensified local host tissue response [1,9]. Mucositis is acknowledged as the principal limiting factor for further treatment intensification in such situations [11]. With the advent of the latest agents being used in combination with radiotherapy, reports of frequent interactions like mucositis are evident in the literature [12].

The first ever method used to determine the effects of radiotherapy on oral mucosa among oral cancer patients was cytologic evaluation, which was reported in 1959 [13,14]. Frequently used scales, based on clinical examination to assess mucositis in CCRT patients, are the World Health Organization (WHO) scale, the National Cancer Institute Common Toxicity Criteria (NCI-CTC), the oral mucositis assessment scale (OMAS) and the Radiation Therapy Oncology Group (RTOG) [4]. All of the mentioned scales primarily assess the oral mucosa for clinical changes, such as erythema and ulceration, and ascribe a score depending on these signs [15]. A commonly used basic scale is the WHO scale, which comprises both objective and subjective measures to determine oral mucositis [16], while the OMAS primarily considers erythema and ulceration at nine different sites around the oral cavity to give a grade for objective measurement. [17]. Therefore, calculating the mature oral epithelial cell percentage in oral smears may be an objective parameter to analyse the consequence of CCRT [4].

Hence, the purpose of this study is the quantification of oral mucositis that progresses during CCRT at the clinical as well as cellular level. In this study, the epithelial cells in oral mucosa will be studied for their viability while comparing them with clinical World Health Organization (WHO) grading and the OMAS assessment scale on the specific days of CCRT. This study also aims to determine the efficacy of the method used, in foreseeing mucositis at earlier stages of CCRT when compared with the WHO and OMAS clinical scoring, which are the current methods in practice.

## 2. Material and methods

### 2.1. Study design and sample characteristics

This was a prospective study, conducted at the Institute of Nuclear Medicine & Oncology Lahore (INMOL), which is an oncology care centre for patients with cancers. Data and samples were collected from the oral squamous cell carcinoma patients presenting for CCRT. The study recruitment period ended when 100 participants had been enrolled as the sample size was calculated with 95% confidence interval by the given formula:

n = Z21-α/2 P (1-P)d2 . 

### 2.2. Participants

Possibly suitable patients were informed in detail about the ongoing study. A total of 100 patients having oral squamous cell carcinoma (OSCC) were selected for the present study and followed up throughout the course of treatment i.e. CCRT. Patients ≥ 18 years old and undergoing CCRT as a treatment option for the first time, patients of both genders and histologically diagnosed with squamous cell carcinoma found at one of the following sites − soft palate, tongue, oropharynx, buccal mucosa, retromolar trigone and floor of mouth were included in the present study. Excluded from the current study were participants who: had previously undergone CCRT; were only on radiotherapy or chemotherapy alone; had received treatment with antibiotics in the 2-week period earlier in the initiation of the treatment; had acute periodontitis or oral candidiasis; had a naso-gastric tube at the initiation of the treatment; and patients with immunocompromised conditions such as diabetes, HIV, etc. 

Radiotherapy was delivered by the use of intensity-modulated radiotherapy treatment (IMRT) and with the use of new fractionation schedules i.e. with concomitant boost and hyperfractionation. Patients who receive IMRT characteristically get one fractionation daily. The patients were planned to receive a total dose of at least 70–119 Gy of radiotherapy given by means of an external beam technique that was evaluated over 7 weeks (1.8–2 Gy/day for 5 days/week). All the targets were receiving the same fraction and dose of radiations i.e. 50−54 Gy, whereby the margins were distinctly enhanced either with concomitant boost or consecutive fractionation schedules. The chemotherapy drug cisplatin and 5-fluorouracil were administered depending on the stage of the tumour [18]. 

For the study group, clinical procedures were conducted on the first day before the start of CCRT, then the next clinical procedure was done on day 5 of treatment, on day 17 of treatment (the midpoint of treatment) and at the end of treatment (CCRT). For the healthy control groups samples were only collected on the first day.

The subjects in the control group were age- and gender- matched and consisted of 30 normal healthy persons (15 male/15 female) who were routinely well, had no addictive habits and were not taking any medication.

### 2.3. Data collection

The demographic records were examined by a trained researcher who gathered data on the following variables, using the designed proforma: age; sex; histological type of malignancy; location of tumour; stage of tumour; history of alcohol and tobacco use; radiation dose; and chemotherapy drug. 

### 2.4. Clinical oral examination

Clinical signs of oral mucositis were noted by means of WHO grading and the OMAS, which are possibly the tools most frequently utilized by clinicians throughout the world. 

Patients who were undergoing CCRT were clinically evaluated for mucositis and scoring was done on the basis of the WHO scale [4] (Table 1). For grading, the buccal mucosa on the treated side and those areas which were included in the radiation therapy were assessed for the mucositis grading.

**Table 1:** WHO grading for oral mucositis.

Grade	Description
Grade 0 (none)	No change
Grade 1 (mild)	Soreness/erythema
Grade 2 (moderate)	Erythema/ ulcers/ can eat solids
Grade 3 (severe)	Ulcers/ requires liquid diet only
Grade 4 (life threatening)	Alimentation not possible

### 2.5. Oral mucositis assessment scale

To obtain OMAS ulceration and erythema scores (total OMAS scores of ulceration/number of sites with ulceration), the nine oral cavity assessed sites were ventral and bilateral tongue, soft and hard palate, upper and lower lip, left and right buccal mucosa and floor of mouth. 

The erythema is evaluated using a 3-point scale as follows: 0 = none (no change in the colour of the mucosa); 1 = mild/moderate (increase in intensity of colour of mucosa) and 2 = severe (mucosa is the colour of fresh blood). 

The OMAS ulceration scoring criteria were as follows:

Grade 0 = no lesion; Grade 1 = <1 cm2; Grade 2 = 1–3 cm2; Grade 3 = >3 cm2.

The value of the OMAS on the respective days of CCRT (before, days 5, 17 and end of CCRT) is obtained by summing the erythema and ulceration scores at each site.

### 2.6. Buccal smear

Before taking buccal smears, every subject was asked to rinse their oral cavity with normal saline. Smears were obtained from the representative sites of buccal mucosa, which were in the field of exposure for the irradiation of malignant tumours and were expected to develop oral mucositis during the CCRT. These smears were taken on respective days of treatment i.e. before the exposure to CCRT, on the 5th day after first exposure to CCRT, on the 17th day of CCRT (the mid-point of therapy) and at 7th week (end of treatment). A wooden spatula was scraped firmly on the buccal mucosa, scrapings were transferred carefully onto frosted glass slides, fixed using alcohol and later stained with Papanicolaou (PAP) stain. A total of four slides were made from each subject on each respective day of sampling and were labeled carefully. Under a light microscope the epithelial cell differentiation and morphology were studied. Epithelial cells were graded as listed in Table 2 [19].

**Table 2:** Maturation stages of epithelial cells on the basis of differentiation and morphology.

Color of epithelial cells	Type of epithelial cells
Orange-stained cells	Mature
Blue/green stained cells	Immature
Partly orange and partly green	Intermediate maturation

### 2.7. Statistical analysis

The data were entered and analysed using descriptive statistics with the aid of the Statistical Package for the Social Sciences (SPSS, version 22.0, IBM Corporation, Armonk, NY, USA). Mean + standard deviation (SD) values were given for quantitative variable like age. Frequencies, percentages and graphs were given for qualitative variables such as oral mucositis grading and epithelial cell keratinization. The data was analyzed by applying the chi-square test and Fisher’s exact tests, and was considered significant if P < 0.05.

## 3. Results 

All patients in this study presented with oral mucositis during the treatment of oral cancers with CCRT, which in a few patients appeared as erythema after a dose of approximately 10 Gy and increased afterwards with a boosted absorption of therapeutic chemo-radiation doses throughout the course of treatment.

**Figure 1:**
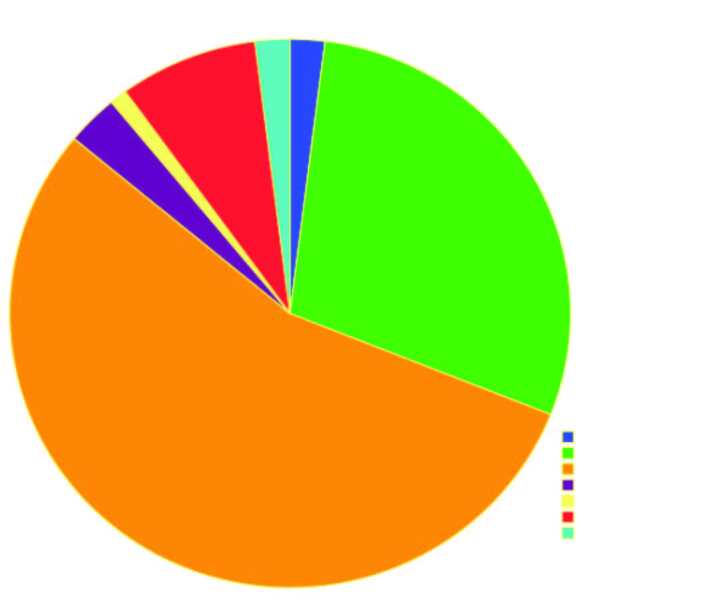
Pie chart showing site distribution of oral squamous cell carcinoma in the study population.

Gender distribution showed that males (62%) were predominant with a male: female ratio of 1.6:1. The patients’ ages ranged from 27 to 80 years with a mean age of 50.21 ± ( SD: 10.647 ). The main stream of patients presented with OSCC involving the tongue (n = 55; 55%) (Figure 1). A majority of the patients presented with the addictive habit of smoking (39%) whereas 39% of patients had no addictive habits. Clinical examination of their oral hygiene revealed that an overwhelming number of patients had poor oral hygiene (51%).

When the OSCC was sub-classified on the basis of their histological subtypes, it was observed that among 100 cases, the majority (98%) were conventional SCC while verrucous carcinoma was seen in only n = 2 (2%) cases. The most common histological grade was moderately differentiated OSCC seen in n = 50 (50%) cases. Similarly, the present study showed that majority of patients receiving CCRT presented with advanced tumour T4 stage (59%).

Considering the fractions of radiotherapy dosages, patients received 70 Gy, 90 Gy and 119 Gy. Half of the patients (50%) received 90 Gy dose of radiotherapy. Similarly, while considering the chemotherapeutic drugs, most of the patients n = 80 (80%) received combination drug therapy (cisplatin and 5-fluorouracil). Table 3 displays the demographic and clinical characteristics of the patients.

**Table 3:** Clinical characteristics of patients of oral squamous cell carcinoma from the INMOL hospital in Lahore (n = 100).

Variable	Value	Confidence interval (95%)
Age		
25−35	n = 11 (11%)	2.67–3.10
36−45	n = 22 (22%)	
46−55	n = 44 (44%)	
56−65	n = 14 (14%)	
65−75	n = 8 (8%)	
76−85	n = 1 (1%)	
Sex		
Male	n = 62 (62%)	1.28–1.47
Female	n = 38 (38%)	
Site		
Lip	n = 2 (2%)	2.79–3.28
Buccal mucosa	n = 29 (29%)	
Tongue	n = 55 (55%)	
Palate	n = 3 (3%)	
Floor of mouth	n = 1 (1%)	
Base of tongue	n = 8 (8%)	
Retromolar area	n = 2 (2%)	
Addictive habits		
Smoking	n =37 (37%)	3.55–4.66
Pan/betel nut + quid	n = 10 (10%)	
Wet snuff/naswar	n = 1 (1%)	
Smoking + pan/betel nut + quid	n = 13 (13%)	
No history	n = 39 (39%)	
Oral hygiene		
Good	n = 5 (5%)	2.34–2.577
Moderate	n = 44 (44%)	
Poor	N = 51 (51%)	
Histological grading		
Well differentiated squamous cell carcinoma	n = 33 (33%)	1.71–2.006
Moderately differentiated squamous cell carcinoma	n = 50 (50%)	
Poorly differentiated squamous cell carcinoma	n = 15 (15%)	
Verrucous carcinoma	n = 2 (2%)	
Clinical staging		
T2	n = 15 (15%)	3.29–3.58
T3	n = 26 (26%)	
T4	n = 59 (59%)	
Surgical treatment		
Yes	n = 21 (21%)	1.70–1.87
No	n = 79 (79%)	
Radiotherapy dosage		
70 Gy	n = 31 (31%)	1.74−2.0
90 Gy	n = 50 (50%)	
119 Gy	n =19 (19%)	
Chemotherapy drugs		
Cisplatin	n = 20 (20%)	2.44–2.75
Cisplatin + fluorouracil	n = 80 (80%)	

All 100 patients who underwent the CCRT treatment had different grading of oral mucosal reaction according to the WHO grading scale and OMAS scale, which were applied to all patients from the first day of the study i.e. first admission for CCRT and followed up till the end of treatment. As the study progressed, the response rate increased from the 17th day until the end of treatment.

**Figure 2:**
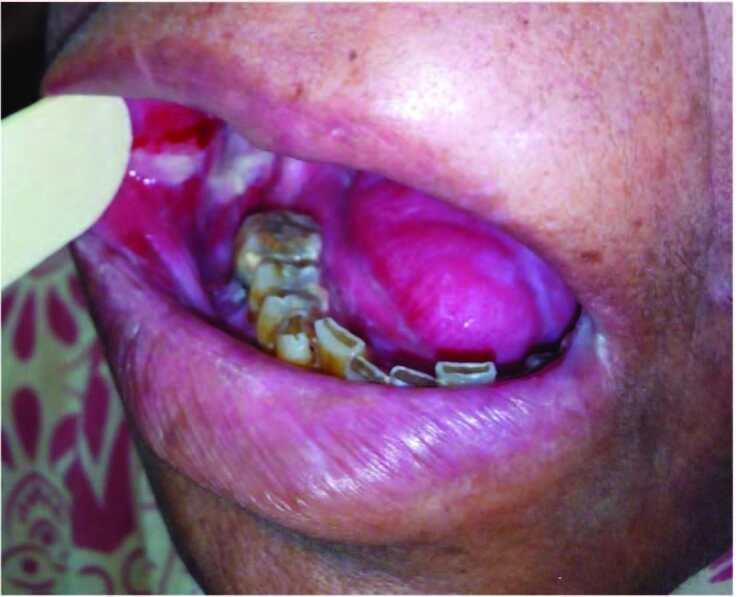
Patient showing WHO and OMAS grade 3 oral mucositis at the end of treatment.

Oral mucositis was observed in all OSCC patients (n = 400) on four different days of CCRT, whereas in control group n = 30 no such changes was observed. According to WHO grading, grade 2 was most commonly seen in n = 86 (21.5%), followed by grade 1 in n = 73 (18.3%), grade 3 in n = 50 (12.5%) and grade 4 in n = 8 (2%). When we compared the WHO oral mucositis grading with the days of treatment, grade 2 was most commonly seen at the mid-point of treatment n = 47 (54.7%), whereas grade 3 and grade 4 were most frequently observed at the end of treatment n = 44 (88%) and n = 8 (100%), accordingly (Figure 2).

For OMAS, the most frequent grade for ulceration observed in the present study was grade 2 n = 116 (29%), followed by grade 3 n = 77 (19%) and grade 1 n = 29 (7.2%); however no lesions were seen in 44.5%, whereas the most frequently noted grade for erythema according to OMAS was grade 1 (34%), followed by grade 2 (19%) and grade 0 (47%), respectively. When we compared OMAS during days of treatment it was noticed that grade 1 was most frequently seen at the 5th day of treatment n = 20 (69%). However, grades 2 and 3 were most commonly reported on the 17th day of CCRT n = 88 (75.9%) and at the end of treatment n = 72 (93.5%), respectively. However, no such findings were seen in the control group.

**Figure 3:**
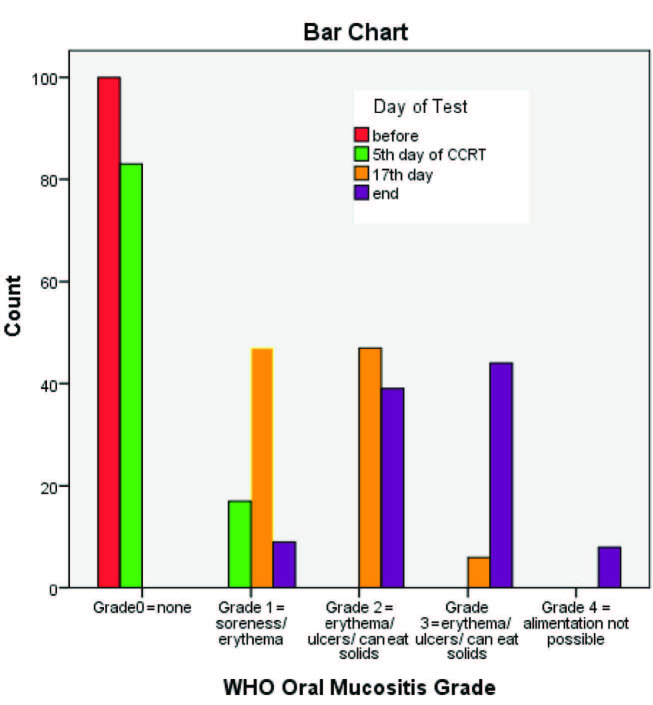
Showing association between WHO oral mucositis grading and days of test/treatment.

When oral mucositis WHO grading and the OMAS scale were compared from day one to the last day of treatment it was noticed that there was a significant rise in the severity and incidence of oral mucositis grading, starting from day 5. A severe type of oral mucositis was developed by the end of treatment, whereas in the middle of the treatment the patients exhibited grade 3, according to WHO, and grade 2, according to OMAS, and none of the patients had grade 0, according to both gradings. By applying a chi-square test, the percentages of WHO grades on different therapy days were significantly different P-value (< 0.0000) and total OMAS scores on different therapy days were significantly different, too (P < 0.0000) (Figure 3). A significant association was also seen between the WHO oral mucositis grading and the age of patient (P < 0.000). Though no statistically significant association was seen among WHO, OMAS and clinical variables but it was noticed that the oral mucositis was higher among males n = 62 (62%) for the WHO grading and the OMAS scale, accordingly. 

**Figure 4:**
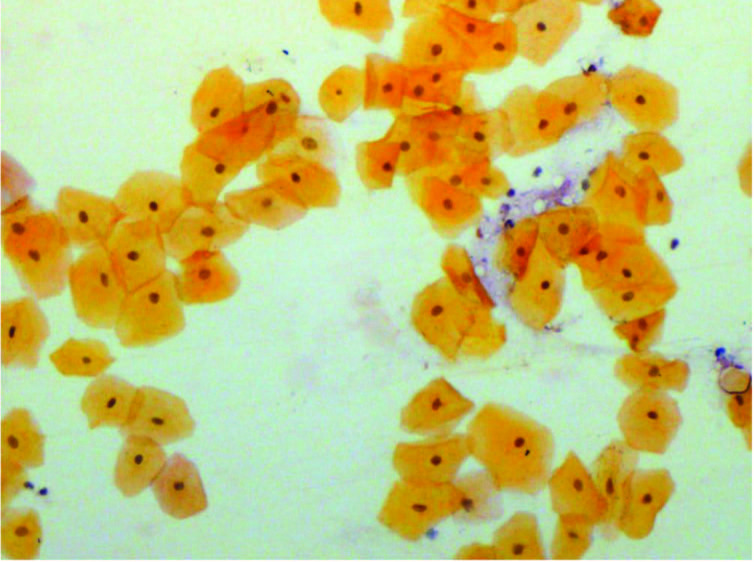
Mature epithelial cells before CCRT stained with PAP (10 ×).

**Figure 5:**
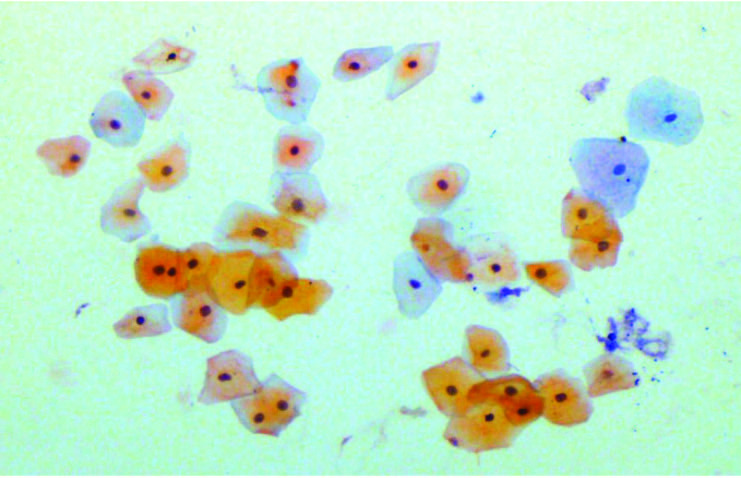
Intermediate and mature cells at the 17th day of CCRT (10 ×).

**Figure 6:**
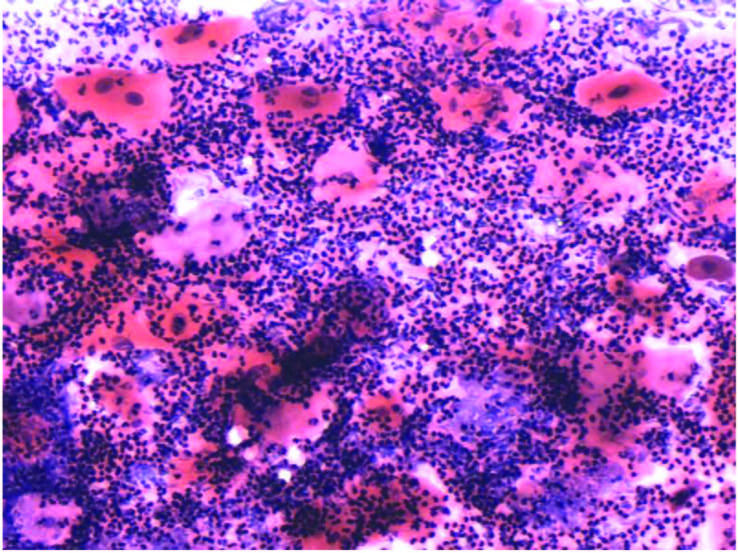
Immature epithelial cells with marked inflammation at the end of CCRT (10 ×).

Out of 400 total smears from 100 patients on different days of CCRT, mature epithelial cells were seen in n = 200 (50%) of smears (Figure 4), whereas intermediate and immature cells were seen in n = 65 (16.3%) and n = 135 (33.8%) (Figures 5 and 6). Whereas in control group, normal cells (large blue, blue-red & red-yellow) were observed on exfoliative cytology. There was statistically significant increase in the percentage of immature cells from the 17th day to the end of CCRT treatment. Among the days of smears, intermediate epithelial cells (64%) were predominantly observed at the 17th day of CCRT, with 95% confidence interval: 2.98−3.04, immature epithelial cells (99%) were most frequently seen at the end of CCRT with 95% confidence interval: 3.65−3.80, whereas mature epithelial cells (100%) were mostly seen before and at the 5th day of CCRT with 95% confidence interval: 1.43−1.56, accordingly. By applying the chi-square test, a significant association (P < 0.0000) was observed among the days of smear and types of epithelial cells (immature, intermediate and mature) and a significant decrease in the percentage of mature epithelial cells was observed from the start to end of CCRT. In addition, a statistically significant association was seen among WHO mucositis grading and types of epithelial cells, also the same findings were observed between OMAS and types of epithelial cells respectively (P < 0.000).

## 4. Discussion

In the present study, it was observed that oral mucositis is predominantly seen in male patients (62%) with a mean age of 50.21, which was in accordance with the study conducted by Igor et al. in 2019 where the mean age was 55 ± 14 years and the prevalence of oral mucositis was higher among males (78.2%) [20]. Previous researchers also found a higher occurrence of oral mucositis in the male population, with approximately 89% and 60%, respectively [21,22]. The greater occurrence in the male population can be described by more incidences of injurious habits ascribed to this gender, such as smoking, use of alcohol, poor hygiene as well as a less frequent visit to dental practitioners [23].

A study carried out in the United States revealed that head and neck tumour patients who received CCRT or cumulative radiation doses of 5000 cGy, are more likely to develop oral mucositis. The finding of that study is similar to the present study [24]. A study conducted in Italy reported that in patients with head and neck cancers, oral mucositis was commonly seen because of the proximity of the oral mucosa to the field of radiation [25]. Furthermore, an association was established between the prevalence of oral mucositis and increased doses of radiation, which is comparable to statistics described in current study. A literature research revealed many similar findings that are in accordance with the present study [26,27].

The OMAS scoring system primarily relies on the measurement of alteration in the oral mucosa (erythema, ulceration). Although the OMAS scale is basic and simple, it requires more time to assess oral mucositis [15,28]. In the current study, based on the OMAS scoring system related to ulceration, the most frequently recorded grade was grade 2 appearing at the end of CCRT. This was in accordance with the study conducted in paediatric cancer patients receiving chemotherapy, which reported that the median maximum site score of OMAS was 1 (interquartile range IQR 0, 2). Moreover, significant correlation was observed between the OMAS and WHO scales which are in concordance with the study conducted in Canada [29]. The validity of OMAS in adults receiving chemotherapy for cancer is well documented. Although OMAS and WHO both appear to be valid, literature reviews have highlighted that the subjective WHO and objective OMAS are delivering different knowledge and it may be significant for future clinical studies to include both scales together.

To overcome the drawbacks of the clinical scoring system, the in vitro assay was also used in the present study. There is increased desquamation of oral epithelial cells as a result of a high dose of radio and chemotherapeutic drugs. In this study, there was statistically significant increase in the percentage of immature cells from the 17th day to the end of CCRT. A statistically significant association was seen between the days of CCRT and type of epithelial cells (P < 0.000), thus suggesting that as CCRT session progressed, the percentage of epithelial viable cells also increased, which was in accordance with the study conducted by Nagarajan stating that the mature cells and immature epithelial cells showed statistically significant decrease and increase (P < 0.0005) from the 2nd week to the 4th week of chemo-radiotherapy, respectively. 

A statistically significant association among the WHO mucositis grading, OMAS and types of epithelial cells (P < 0.000) was also noticed in this study, which was in contrast with the study conducted by Nagarajan showing that in the 2nd week there was a significant rise in viable cells compared to the WHO score [30].

## 5. Conclusion

The present study’s results reveal a significant association among the evaluation of the CCRT-induced oral mucositis, using the WHO, OMAS scale and in vitro assessment in patients with advanced oral cancer. All of these are valuable means for enhancing the clinical evaluation of oral mucositis. For those patients where oral examination is not possible, the WHO and OMAS scales can be implemented in such conditions.

The oral mucositis can be critical with the progression of CCRT, affecting the quality of life and interfering with CCRT treatment as well. It is suggested that multidisciplinary teams and patients must discuss the severity and onset of CCRT-induced oral mucositis therefore providing the best supportive care to patients suffering from CCRT- induced oral mucositis.

## Informed consent

This study has been approved by the Advanced Studies & Research Board (ASRB) of the University of Health Sciences, Lahore and written informed consent was obtained from all study participants before the start of the clinical procedure.
